# Aerial Phenotyping of Coffee Flowering Using Deep Learning for Early Yield Prediction

**DOI:** 10.3390/plants15193019

**Published:** 2026-10-02

**Authors:** Elias Horácio Zavala, Gabriel Araújo e Silva Ferraz, Felipe Andrés Obando Vega, Mirian de Lourdes Oliveira, Milene Alves de Figueiredo Carvalho, Samuel Henrique Braga da Cunha, Andrea Pagliai, Daniele Sarri

**Affiliations:** 1 Department of Agricultural Engineering (DEA), School of Engineering (EENG), Federal University of Lavras (UFLA), Lavras 37203-202, MG, Brazil; elias.zavala1@estudante.ufla.br (E.H.Z.); olivemiri@gmail.com (M.d.L.O.); 2 Faculty of Engineering and Agricultural Sciences, Digital University Institution of Antioquia (IU Digital), Medellín 050015, Antioquia, Colombia; felipe.obando@iudigital.edu.co; 3 Embrapa Café, Empresa Brasileira de Pesquisa Agropecuária (Embrapa), Brasília 70770-901, DF, Brazil; milene.carvalho@embrapa.br; 4 Department of Agriculture, Food, Environment and Forestry (DAGRI), University of Florence, 50145 Florence, Italy; samuelhbraga@gmail.com (S.H.B.d.C.); andrea.pagliai@unifi.it (A.P.); daniele.sarri@unifi.it (D.S.)

**Keywords:** *Coffea arabica*, aerial phenotyping, Deep Learning, remotely piloted aircraft, yield prediction

## Abstract

Early yield estimation is important for improving decision-making and management practices in coffee production systems. However, approaches that use aerial imagery to characterize flowering and relate it to subsequent yield remain limited. This study evaluated the potential of RGB imagery acquired by remotely piloted aircraft (RPA) combined with Deep Learning techniques to classify coffee plants according to three levels of a flowering proxy and to investigate its relationship with yield. A dataset comprising 1593 segmented RGB images was classified into Low, Medium, and High flowering levels using a custom convolutional neural network and three transfer learning architectures: VGG16, ResNet50V2, and MobileNetV2. ResNet50V2 was selected for final evaluation based on its validation performance and achieved 92.12% accuracy, 92.40% macro-precision, 91.81% macro-recall, and 92.06% macro-F1 on the test dataset. The flowering proxy showed a positive association with field-observed yield, with Spearman’s ρ = 0.701 (*p* < 0.001) and R^2^ = 0.528 for the combined dataset of Arara and Mundo Novo. In consolidated out-of-sample predictions, the model achieved R^2^ = 0.489, MAE = 1.03 kg plant^−1^, and RMSE = 1.18 kg plant^−1^. Performance differed between cultivars, with consolidated R^2^ values of 0.567 for Arara and 0.329 for Mundo Novo. Spatial analysis also showed correspondence between the distribution of flowering levels derived from RGB imagery and the subsequent spatial distribution of yield. These findings indicate that RPA-based RGB imagery and Deep Learning can provide spatialized information related to coffee flowering and its productive performance, with potential applications in precision agriculture. Further evaluation across production cycles, cultivars, and environmental conditions is needed to assess the robustness and generalization of the approach.

## 1. Introduction

Coffee production is one of the main agricultural activities in Brazil, playing a fundamental role in the economy of several producing regions. The country remains the world’s largest producer and exporter of coffee, with an estimated production of 58.9 million bags in the 2024/25 season [1]. In addition to its economic importance, coffee farming contributes to employment generation and regional development [2].

Accurate yield forecasting is essential for improving crop management, optimizing resource allocation, and supporting decision-making. Among the phenological stages of coffee, flowering is recognized as one of the main indicators of yield potential, since the number and intensity of flowers are directly associated with fruit set and final production [3,4]. However, flowering assessment is still predominantly performed through visual field observations, a labor-intensive, time-consuming, and inherently subjective method.

Recent advances in precision agriculture have expanded the use of remotely piloted aircraft (RPA) equipped with high-resolution sensors for agricultural monitoring. Compared with satellite imagery, RPAs provide higher spatial and temporal resolution, as well as greater acquisition flexibility, characteristics that are particularly relevant for monitoring coffee flowering, which occurs over short and dynamic periods [5]. Previous studies have demonstrated the applicability of RPA imagery for assessing plant health, estimating biomass, and characterizing spatial variability in coffee plantations [6,7,8,9].

Similarly, Deep Learning techniques, particularly Convolutional Neural Networks (CNNs), have been increasingly used to extract information from agricultural imagery. In coffee production systems, CNN-based approaches have been employed to identify and count coffee plants using RGB imagery, achieving accuracies close to 97% [10]. Likewise, artificial intelligence models have been used for crop yield prediction based on climatic and management variables, showing performance comparable to or higher than traditional statistical methods [11,12].

Despite these advances, some studies have explored the use of digital imagery and computer vision techniques to characterize coffee flowering, including approaches based on Convolutional Neural Networks [13] and RGB imagery acquired by UAVs [14,15,16]. However, studies integrating flowering information obtained from RPAs with Deep Learning models for coffee yield prediction remain limited. In particular, the combined use of flowering intensity and its spatial distribution as predictive variables for coffee yield remain relatively unexplored.

Therefore, this study evaluated whether flowering intensity and its spatial distribution obtained from RPA imagery can be used as predictive variables for coffee yield using Deep Learning models. We hypothesize that flowering intensity is positively associated with final yield, enabling earlier estimates of coffee production. The proposed approach aims to provide a tool for aerial phenotyping and yield forecasting in coffee production systems.

## 2. Materials and Methods

**Figure 1:**
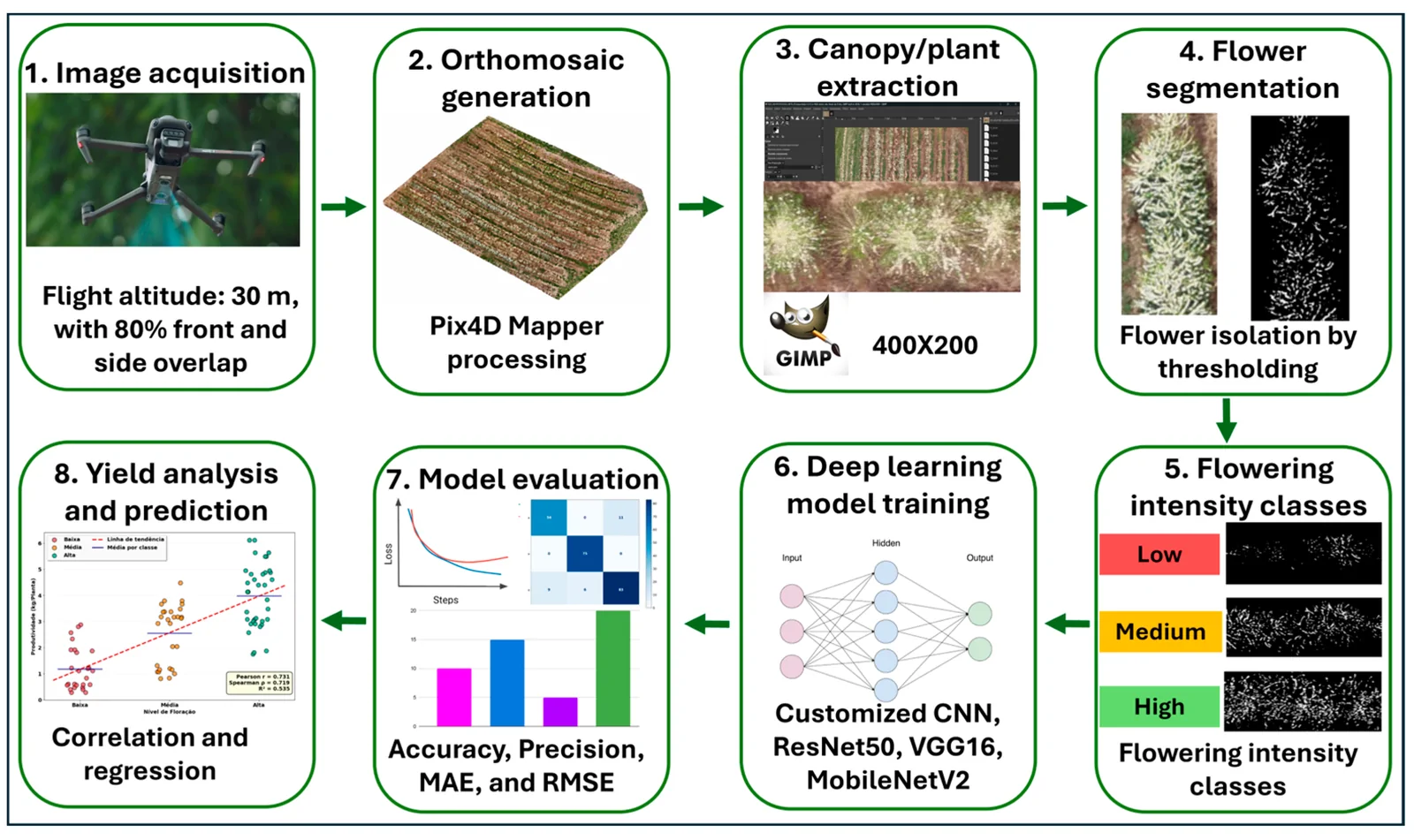
Methodological framework.

The methodological framework of this study comprised eight sequential stages, ranging from aerial image acquisition to coffee yield prediction, as illustrated in Figure 1. Initially, RGB images were acquired using remotely piloted aircraft (RPA) and processed to generate orthomosaics. Subsequently, individual plant canopies were extracted and subjected to flower segmentation using thresholding, allowing the classification of flowering intensity into three classes (low, medium, and high). Different Deep Learning models were then trained and evaluated for the automatic classification of these flowering classes. Finally, the predicted classes were related to the observed yield through correlation and regression analyses to assess the potential of flowering intensity as a phenotypic indicator for early coffee yield prediction.

### 2.1. Description of the Study Area

**Figure 2:**
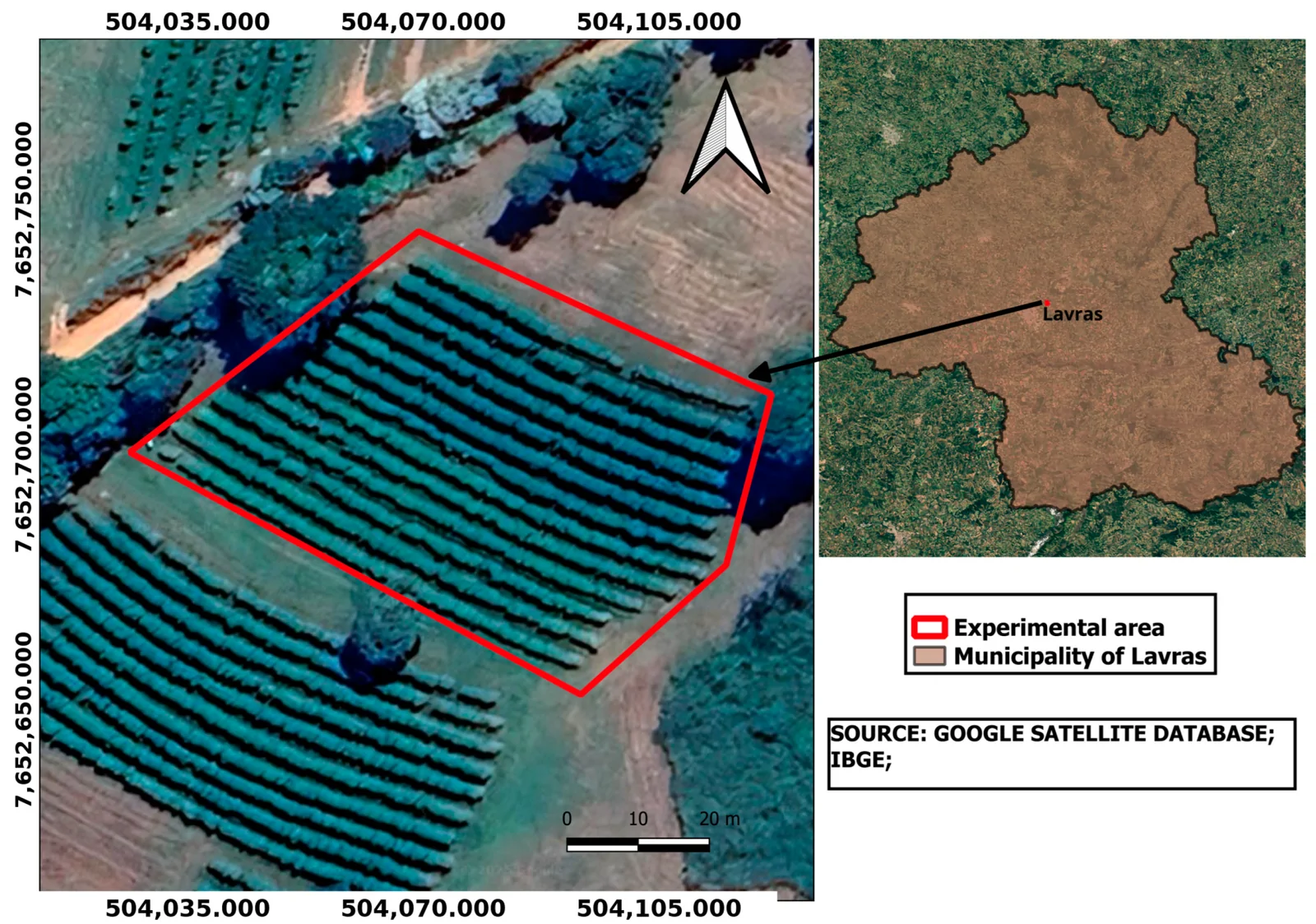
Location of the study area.

The experiment was conducted at the experimental area of the Coffee Sector of the Department of Agriculture at the Federal University of Lavras (UFLA), in Lavras, Minas Gerais, Brazil (Figure 2). The area was cultivated with six-year-old *Coffea arabica* L. plants of the Mundo Novo 376-4 and Arara cultivars, established at a spacing of 3.60 m between rows and 0.60 m between plants. The region has a tropical highland climate (Cwa, Köppen classification), characterized by dry winters and rainy summers, with an average annual temperature of 20.4 °C, maximum temperatures close to 28 °C, minimum temperatures of 12 °C, and an average annual precipitation of approximately 1530 mm, concentrated between October and March.

### 2.2. Data Collection

The images were acquired using two remotely piloted aircraft (RPA) systems: a DJI Mavic 3T Enterprise equipped with a 20 MP RGB camera and a 3DR Solo equipped with a Parrot Sequoia multispectral camera, from which only the 16 MP RGB sensor was used. The use of RGB sensors from different platforms enabled the composition of a dataset with greater diversity in acquisition characteristics, while the exclusive use of RGB images maintained the same type of spectral information across all samples.

Four flights were conducted on 10 October 2024, with two flights performed using each RPA system. Flights were conducted between 11:00 and 12:00 h, at an altitude of 30 m, a flight speed of 5 m s^−1^, and 80% front and side overlap. After acquisition, the image datasets were processed in Pix4D Mapper (version 4.5.6) to generate the orthomosaics, using the default photogrammetric processing parameters. The resulting orthomosaics were subjected to the same processing workflow and image extraction procedure.

Each orthomosaic was manually cropped using GIMP (GNU Image Manipulation Program, version 3.0.2-1), using fixed windows of 400 × 200 pixels at a spatial scale equivalent to an experimental plot composed of five coffee plants. The standardized crop dimensions provided a common matrix structure for the image samples used in the classification models. A total of 1593 image crops were generated for the classification dataset.

For the analyses involving field-measured yield, a separate set of 90 image crops was obtained, with each image corresponding to an experimental plot composed of five coffee plants for which yield was determined in the field. This dataset comprised 45 plots of Mundo Novo and 45 plots of Arara and was used for the validation and for the correlation and regression analyses between image-derived information and field-measured yield.

No individual comparison between the platforms was performed, as the objective was to evaluate flowering intensity classification from RGB images under the variability present in the dataset.

All image processing and computational model development steps were performed in Python (version 3.11.7) using the Google Colab environment. The OpenCV library was used for image processing, NumPy for matrix operations, Pandas for data manipulation, TensorFlow/Keras for implementing the Deep Learning models, Scikit-learn for model training and performance metric calculation, and Matplotlib for result visualization.

### 2.3. Data Processing

**Figure 3:**
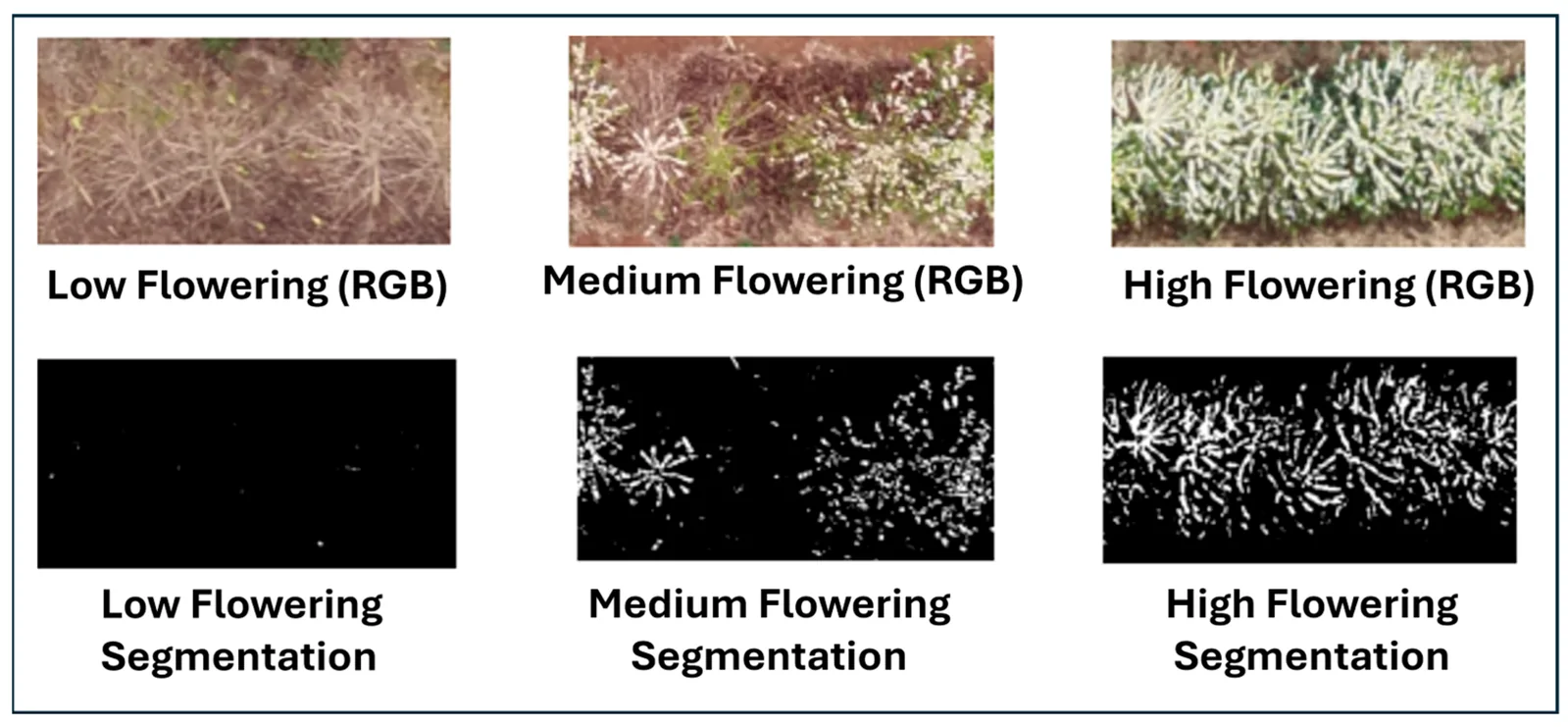
Representative images of the flowering classes obtained through color segmentation (fixed threshold of 215, grayscale).

For the generation of the proxy labels, the RGB images were converted to grayscale using the ‘cvtColor’ function from OpenCV. Subsequently, binary thresholding was applied using 215 as the reference threshold. Pixels with intensity values greater than 215 were assigned a value of 255 (white), whereas pixels with intensity values equal to or lower than 215 were assigned a value of 0 (black). The number of white pixels was then determined for each image and used as an indirect indicator of flowering intensity (Figure 3).

A value of 215 was adopted as the reference threshold for the classification procedure. Since the choice of a threshold can influence the number of pixels classified as white and, consequently, the definition of the classes, a sensitivity analysis was performed using values of 210, 215, and 220. This analysis allowed the stability of the classifications to be assessed under small changes in the adopted threshold, as described in Section Threshold Sensitivity Analysis.

**Figure 4:**
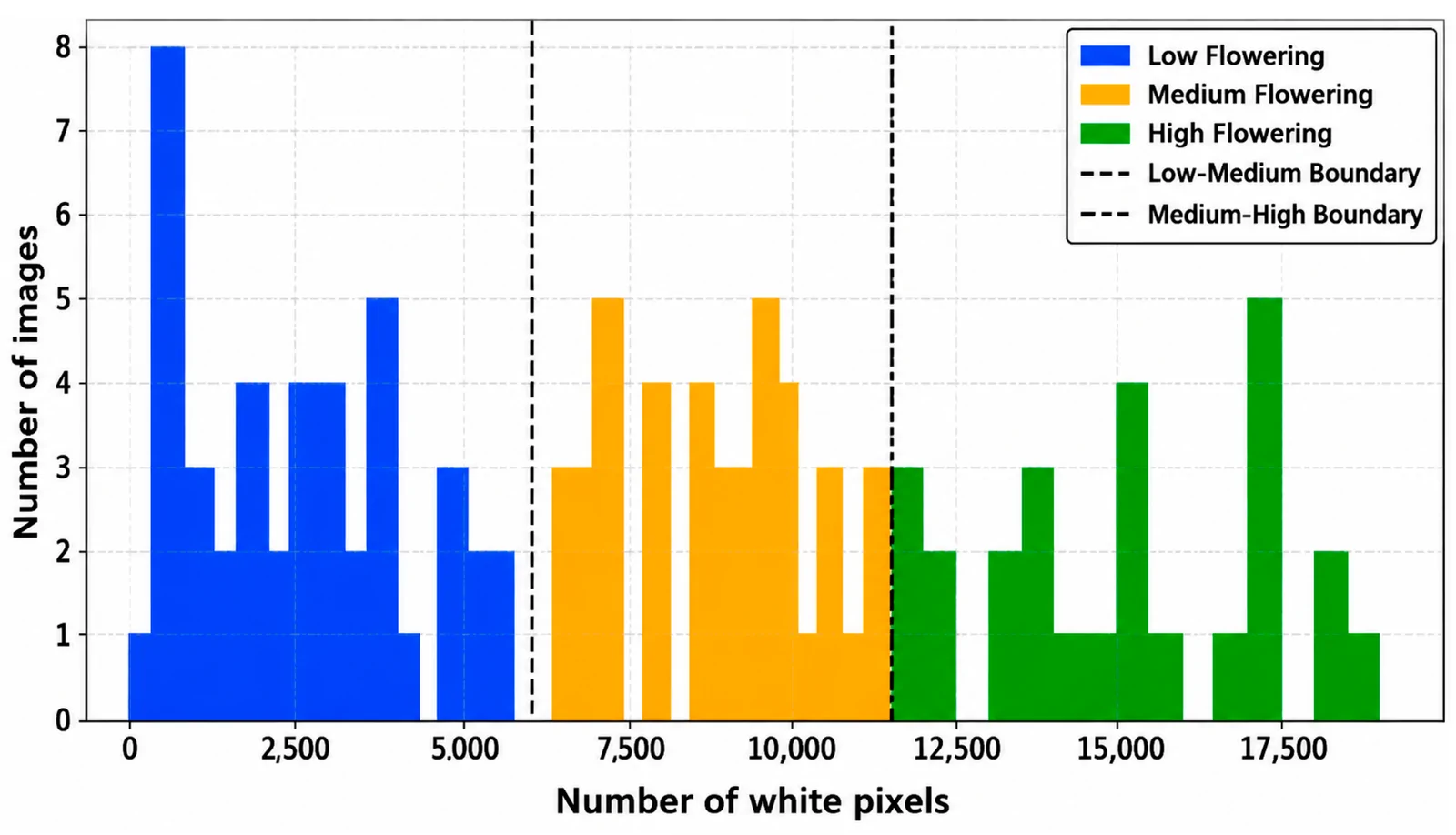
Distribution of white pixels (flowers).

The flowering-related classes, hereafter referred to as flowering proxy classes, were defined based on the distribution of the number of white pixels obtained using the reference threshold (Figure 4). The minimum and maximum values observed in the dataset were identified, and the range between these values was divided into three intervals of equal width. The images were then classified as Low, Medium, or High flowering intensity according to the corresponding interval.

The resulting classes were considered proxy categories of flowering intensity because they were derived from the number of pixels classified as white rather than from a direct count of individual flowers. Thus, the procedure provides a reference variable based on the spectral response of the images without assuming that each white pixel necessarily corresponds to a flower.

After class assignment, the original RGB images were organized into their respective classification directories. The binarized images and white-pixel counts were used exclusively to generate the proxy labels. The original RGB images, without thresholding, were used as input to the Deep Learning models.

#### Threshold Sensitivity Analysis

To assess the sensitivity of the definition of the proxy classes to the adopted intensity threshold, additional classifications were performed using values of 210 and 220, in addition to the reference threshold of 215. For each threshold, the binary image was generated, the number of white pixels was determined, and the range between the minimum and maximum values was divided into three intervals of equal width, resulting in the Low, Medium, and High classes.

The classifications obtained with the different thresholds were compared using percentage agreement and Cohen’s kappa coefficient. Additionally, the numbers of white pixels obtained with each threshold were compared using Pearson’s correlation coefficient. These analyses were used to assess the stability of the classifications and the number of white pixels in response to small changes in the adopted threshold.

A deterministic thresholding and counting baseline was included as a procedural reference for the Deep Learning models. For each image, the number of white pixels obtained using the reference threshold of 215 was directly assigned to the Low, Medium, or High classes using the same boundaries employed to generate the proxy labels.

Because the baseline directly applies the same rule used to generate the proxy classes, high agreement with the labels is expected. Therefore, it was used as a deterministic procedural control to establish the expected performance when the label-generation rule is applied directly, rather than as an independent machine learning model. The baseline and the configurations of the Deep Learning models are presented in Table 1.

### 2.4. Deep Learning Classification

The Deep Learning models were trained using the original RGB images, without applying the thresholding procedure used to generate the proxy labels. The images, with dimensions of 400 × 200 pixels and three color channels (400 × 200 × 3), were directly used as model inputs and normalized by dividing the pixel values by 255.

The dataset was randomly divided into training, validation, and test sets in proportions of 70%, 15%, and 15%, respectively, using a fixed random seed of 88. The test set was kept separate from the training process and used exclusively for the final evaluation of the models.

During training, data augmentation was applied to increase the variability of the images used by the models. The procedure included horizontal and vertical flips, rotation of approximately ±30°, zoom of up to 20%, horizontal and vertical translations of up to 10%, 20% contrast adjustment, and 20% brightness adjustment.

Four Deep Learning architectures were evaluated: a custom CNN, VGG16, MobileNetV2, and ResNet50V2. The architectural characteristics, training strategies, and learning rates of each model are presented in Table 1. All models performed classification into three categories corresponding to the proxy flowering intensity classes: Low, Medium, and High.

For the custom CNN, VGG16, and MobileNetV2, training was performed using the Adam optimizer, with learning rates of 0.001, 0.0001, and 0.0001, respectively, and a maximum limit of 150 epochs. Early stopping was configured with a patience of 25 epochs, while the learning rate was reduced by a factor of 0.30 after eight epochs without improvement. For ResNet50V2, the two-stage training strategy described in Table 1 was employed, with a maximum limit of 50 epochs in the first stage and 100 epochs in the second stage, including the initial freezing of the base network followed by fine-tuning of the final layers.

Model performance was evaluated using the independent test set. Overall accuracy, weighted precision, weighted recall, and weighted F1-score were calculated. Confusion matrices and per-class classification reports were also generated. The Deep Learning models were compared with the deterministic thresholding and counting baseline as a reference for the procedure used to construct the proxy labels.

The use of the original RGB images as input allowed the models to perform classification without explicitly applying, during inference, the steps of grayscale conversion, thresholding, and white-pixel counting. However, because the target classes were defined based on this procedure, the Deep Learning models were evaluated as classifiers of proxy flowering intensity categories rather than as direct estimators of the actual number of flowers. Therefore, the results were interpreted in terms of the consistency of the classification of these proxy categories.

### 2.5. Model Validation and Evaluation

Model validation and evaluation were performed using yield data obtained at harvest, with the objective of assessing the relationship between the flowering proxy obtained from the images and coffee yield. The analysis comprised 90 experimental plots, each consisting of five coffee plants. The plot was considered the experimental unit and, therefore, the unit of analysis. Yield was obtained for each plot and subsequently divided by the number of plants (five) to express yield as kg plant^−1^. Thus, the dataset used in the analyses comprised 90 independent observations corresponding to the 90 experimental plots, with each observation containing the flowering proxy class and the corresponding yield per plant. The data were organized according to experimental-plot identification, flowering proxy class, and observed yield. Observations with missing values for the flowering proxy or yield were excluded from the analyses.

The flowering proxy was represented by three ordered classes low flowering, medium flowering, and high flowering obtained from the image-processing procedure described previously. These classes were numerically coded as 1, 2, and 3, respectively. This coding preserved the ordered structure of the proxy classes and allowed their inclusion as an explanatory variable in the statistical analyses. This classification was used as an indirect indicator related to flowering and did not constitute a direct measure of flowering intensity or the quantity of flowers present on the plants.

Differences in yield among the flowering proxy classes were assessed using the Kruskal–Wallis test. Pairwise comparisons were subsequently performed using the Mann–Whitney U test, with Bonferroni correction applied to account for multiple comparisons. Descriptive statistics were also calculated for each flowering proxy class.

The monotonic association between the flowering proxy and yield was evaluated using Spearman’s rank correlation coefficient (ρ). In addition, a simple linear regression model was fitted, with yield as the response variable and the ordinal flowering proxy classification as the explanatory variable. The regression coefficient, its 95% confidence interval, statistical significance, and coefficient of determination (R^2^) were used to characterize the relationship between the flowering proxy categories and yield.

To distinguish the explanatory fit of the regression model from its predictive performance, repeated 5-fold cross-validation was performed with 10 repetitions, resulting in 50 training and testing evaluations. In each iteration, the regression model was fitted exclusively using the training observations and subsequently used to predict yield for observations that were not included in model fitting. Thus, R^2^, mean absolute error (MAE), and root mean square error (RMSE) represented out-of-sample predictive performance. MAE quantified the mean absolute difference between observed and predicted yield, whereas RMSE provided a measure that assigned greater weight to larger prediction errors. Both error metrics were expressed in kg plant^−1^.

The mean and standard deviation of R^2^, MAE, and RMSE across the 50 cross-validation evaluations were calculated to characterize the stability of model performance across the different resampling procedures. In addition, the predictions generated for each plant across the different validation stages were averaged to obtain consolidated out-of-sample predictions, from which consolidated R^2^, MAE, and RMSE were calculated.

## 3. Results

The sensitivity analysis of the 210, 215, and 220 thresholds showed different levels of agreement among the proxy flowering-intensity classes (Table 2). The comparison between 210 and 215 resulted in 94.22% agreement, with a Cohen’s Kappa coefficient of 0.908. For the 215 and 220 thresholds, the agreement was 93.72%, with a Kappa coefficient of 0.900. The comparison between the 210 and 220 thresholds resulted in 87.95% agreement, with a Kappa coefficient of 0.809.

The results indicated agreement among the classifications obtained with the different threshold values. The comparisons involving the 215 threshold showed agreement values above 93%, whereas the comparison between the extreme thresholds resulted in 87.95% agreement.

The variation observed among the evaluated thresholds indicates that the proxy classes underwent limited changes in response to small variations in the threshold value. Therefore, the value of 215 was maintained for the subsequent analyses.

### 3.1. Performance of Deep Learning Architectures in Classifying the Flowering Proxy

The threshold-based baseline achieved 100% classification accuracy, as expected from the deterministic rule used to construct the flowering proxy categories. As described in the methodology, the same thresholding procedure was used to derive the class labels; therefore, its complete agreement reflects the consistency of the rule-based classification with the labels generated by that procedure, rather than an independent measure of classification performance. Similar threshold-based approaches have been used for coffee flower characterization, including the binarization procedure described by [13], who combined threshold-based extraction with a CNN to characterize coffee flowers under different image acquisition conditions.

The performance of the Deep Learning architectures evaluated for classification of the flowering proxy is presented in Table 3. Architecture selection was performed based on the validation set, considering the macro F1-score, in order to avoid using the test set during model selection.

Among the evaluated architectures, ResNet50V2 presented the highest macro F1-score on the validation set (0.9443), followed by MobileNetV2 (0.9305), Custom CNN (0.8467), and VGG16 (0.8332). ResNet50V2 also presented the highest validation accuracy (94.54%), macro precision (0.9411), and recall (0.9479), in addition to the lowest loss value (0.1342). The use of macro F1-score as one of the main selection metrics allows precision and recall across the different classes to be considered jointly, avoiding an evaluation determined solely by the performance of a specific class.

The performance observed for ResNet50V2 indicates that the architecture was able to learn visual features capable of differentiating the three categories established by the proxy. This behavior is consistent with the ability of deep convolutional networks to extract hierarchical features from images. In residual networks, shortcut connections were introduced to facilitate the optimization of deep networks and promote the learning of residual representations [17]. Thus, the observed performance may be related to the architecture’s ability to represent visual differences among the proxy categories, although this relationship does not allow the result to be attributed exclusively to the residual structure of the network.

Although MobileNetV2 showed performance close to that of ResNet50V2, with an accuracy of 93.28% and a macro F1-score of 0.9305, ResNet50V2 presented slightly higher values for the metrics considered in the selection. Therefore, ResNet50V2 was selected for the final evaluation, without implying a marked performance difference relative to MobileNetV2. The ResNet architecture uses residual connections, whereas MobileNetV2 employs inverted residuals and depthwise convolutions and was also developed with an emphasis on computational efficiency [17,18].

The difference between the two architectures was relatively small, with a difference of 1.26 percentage points in validation accuracy and 0.0138 in macro F1-score. This result indicates that both architectures were able to learn sufficient features to distinguish the proxy categories under the evaluated conditions. Thus, the selection of ResNet50V2 was based on the validation metrics adopted in this study rather than on a broad performance difference relative to MobileNetV2.

After selection, the test set was used only for the final evaluation of ResNet50V2. On this set, the model achieved 92.12% accuracy, 0.9240 macro precision, 0.9181 macro recall, and 0.9206 macro F1-score, with a loss of 0.2814. The reduction relative to the values observed during validation indicates a difference in performance between the datasets, which is expected when the model is evaluated on observations that did not participate in architecture selection. The maintenance of values close to or above 0.90 for the main metrics indicates that the model maintained similar performance between validation and testing for the categories defined by the proxy.

The use of visual information to characterize aspects related to coffee flowering has also been explored in previous studies. Ref. [13] for example, combined binarization and convolutional neural networks to identify coffee flowers in digital images. The authors observed that information related to pixels and the spatial characteristics of images could contribute to flower identification, although performance varied according to illumination conditions and acquisition angle. In the present study, visual information was used for a different purpose, since the bright pixels served as the basis for establishing flowering proxy categories rather than for the individual identification of flowers.

The results obtained for Custom CNN and VGG16 were lower than those observed for ResNet50V2 and MobileNetV2 during validation, with macro F1-scores of 0.8467 and 0.8332, respectively. These differences indicate that, under the conditions of this study, ResNet50V2 and MobileNetV2 showed greater capacity to reproduce the categories established by the proxy. However, these differences should be interpreted within the specific context of the image dataset, preprocessing, and training procedure employed, and are not sufficient to establish the general superiority of one architecture over another based solely on these results.

### 3.2. Performance of ResNet50V2 in Classifying the Flowering Proxy

**Figure 5:**
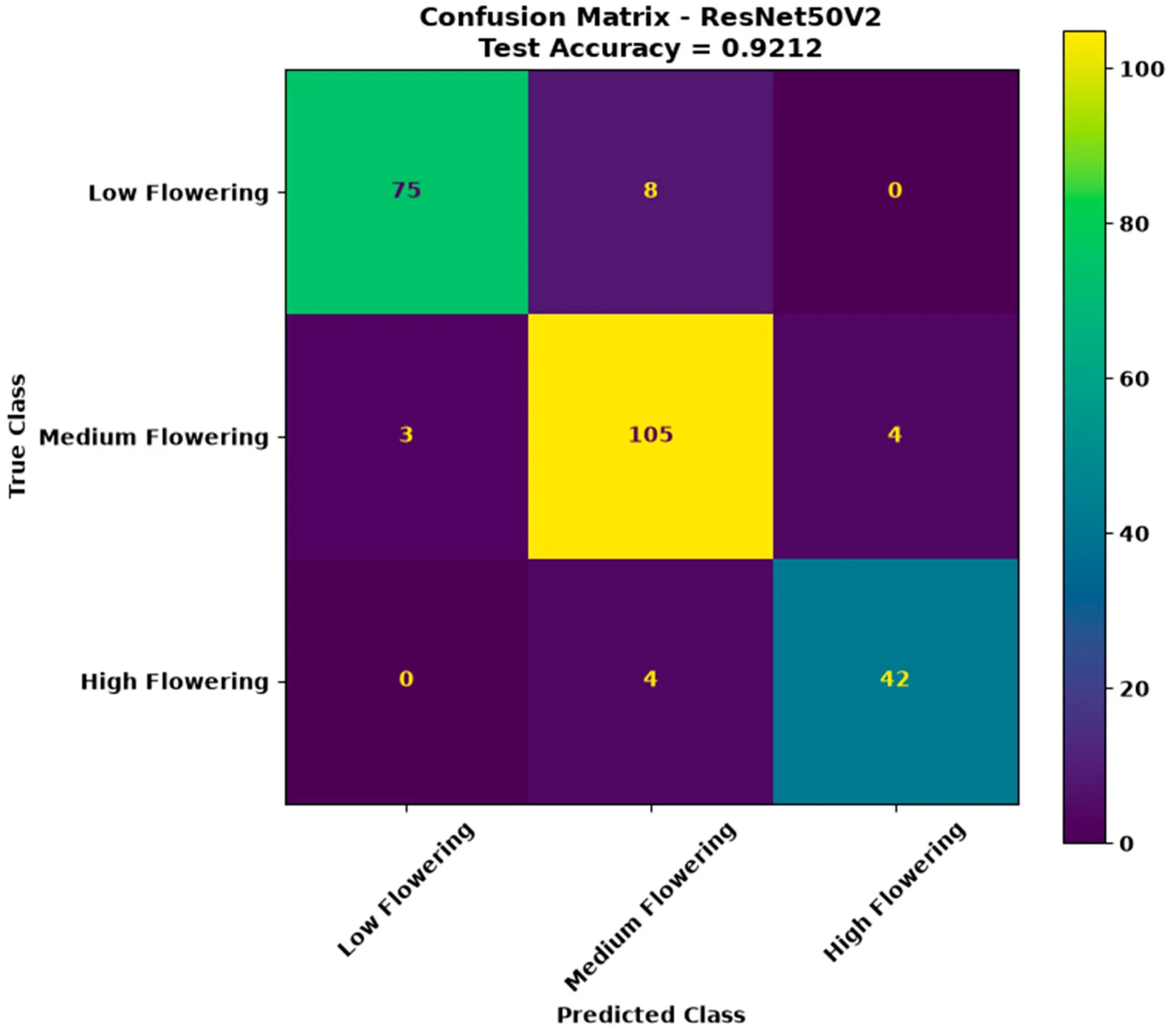
Confusion matrix of the ResNet50V2 model for classification of the flowering proxy on the test set.

The confusion matrix of ResNet50V2 on the test set is presented in Figure 5. Of the 241 images evaluated, 222 were correctly classified, resulting in an accuracy of 92.12%. Among the classes, 75 correct classifications were observed for Low, 105 for Medium, and 42 for High.

Analysis of the confusion matrix showed that classification errors occurred exclusively between adjacent classes of the flowering proxy. For the Low class, 75 of the 83 observations were correctly classified, while 8 were classified as Medium. For the Medium class, 105 of the 112 observations were correctly classified, with 3 classified as Low and 4 as High. For the High class, 42 of the 46 observations were correctly classified, and 4 were classified as Medium. No direct classifications between the Low and High classes were observed in either direction.

This pattern indicates that the main classification difficulties occurred in distinguishing consecutive levels of the flowering proxy, while no errors directly involving the extreme categories were observed. The greater occurrence of errors between Low–Medium and Medium–High suggests greater visual similarity between consecutive proxy categories. Because these categories were established based on the amount of bright pixels identified in the images, small differences in this characteristic may result in observations belonging to different categories but remaining visually similar.

This behavior is also relevant in light of results reported in the literature for coffee flower identification. Ref. [13] observed that image characteristics, including illumination conditions and acquisition angle, can influence the identification of floral structures. The authors also highlighted the possibility of confusion between flowers and background elements, particularly under certain acquisition conditions. In the present study, although the task was different, image crops were performed in areas of interest to minimize the influence of external elements and potential sources of noise. However, this strategy does not completely eliminate the possibility of influence from background characteristics, illumination, and other elements present in the images. Thus, visual similarity between consecutive proxy levels may contribute to the confusions observed between adjacent categories.

Considering the metrics calculated from the confusion matrix, the Low class presented a precision of 0.9615, recall of 0.9036, and F1-score of 0.9317. For the Medium class, the values were 0.8974, 0.9375, and 0.9170, respectively, whereas the High class presented precision of 0.9130, recall of 0.9130, and F1-score of 0.9130. The similarity of these values among the three categories contributed to the macro F1-score of 0.9206 observed on the test set.

The distribution of the class-specific metrics indicates that none of the categories presented substantially lower performance than the others. The Low class presented the highest precision, whereas the medium class presented the highest recall, indicating that most observations belonging to this category were correctly identified. For the High class, precision and recall presented the same value, indicating a balance between correct classifications and errors associated with this category. Thus, the overall performance does not appear to have been determined exclusively by one of the classes.

It should be emphasized, however, that classification was based on a flowering proxy obtained from image processing, based on the amount of bright pixels identified in the analyzed areas. Thus, the Low, Medium, and High categories represent different levels of occurrence of bright pixels potentially associated with the presence of floral structures in the image, rather than an individual count of flowers present on the plants. Therefore, the performance of ResNet50V2 indicates the ability of the architecture to reproduce, from the images, the categories defined by this visual indicator, whose variation is related to the amount of bright pixels observed.

This approach presents an important difference from studies that also use RGB images to characterize flowering. Ref. [14] used the area occupied by flowers in the images as a measure related to flowering and established its relationship with the number of flowers obtained through field counting. Similarly, ref. [19], working with UAV RGB imagery in apple orchards, used flower-associated pixels extracted through image thresholding to derive indices related to flowering intensity. In the present study, the area represented by the bright pixels was used differently, as the basis for establishing categories of the flowering proxy, without converting this information into the number of flowers or a continuous flowering index. Thus, these studies share the use of image-derived information associated with floral structures but differ in how this information is transformed and interpreted. Whereas [14] related the floral area identified in coffee images to the number of flowers observed in the field, and [19] derived flowering indices from flower-associated pixels in apple trees, the present study used the occurrence of bright pixels to define categorical levels of the flowering proxy.

This distinction also helps delimit the interpretation of the results obtained with ResNet50V2. The model was developed to reproduce the categories established by the proxy from the images, rather than to directly estimate the number of flowers present on the plants. Therefore, the observed performance should be interpreted in the context of the classification of this visual indicator, whereas the relationship between the pixels associated with floral structures and the actual number of flowers remains an issue requiring specific evaluation in future studies using independent field measurements.

### 3.3. Flowering–Yield Relationships in Coffee

The combined analysis of the Arara and Mundo Novo cultivars indicated a positive association between flowering level and plant yield. Spearman’s correlation showed a coefficient of ρ = 0.701 (*p* < 0.001), indicating a positive monotonic association between the variables. In the linear regression, flowering level was treated as an ordinal variable (1 = Low, 2 = Medium, and 3 = High). The model, including cultivar, flowering level, and their interaction, presented an R^2^ = 0.528 and an adjusted R^2^ = 0.511. Flowering level was positively associated with yield (β = 1.43 kg plant^−1^ per increment in flowering level; 95% CI: 1.08–1.79; *p* < 0.001). A significant flowering level × cultivar interaction was also detected (β = −0.52; 95% CI: −1.02 to −0.02; *p* = 0.042), indicating that the magnitude of the association between flowering level and yield differed between cultivars. The estimated slope was 1.43 kg plant^−1^ per flowering-level increment for Arara and 0.91 kg plant^−1^ for Mundo Novo.

The positive relationship observed is biologically consistent with previous results relating flowering characteristics to coffee yield. Ref. [20], when evaluating phenotypic traits of *Coffea arabica* L., identified associations between plant characteristics, including flower number and yield, highlighting the relevance of these traits in explaining yield variability. Complementarily, ref. [4] evaluating different Arabica coffee harvests in southern Minas Gerais and Alto Paranaíba, found associations between yield and phenological characteristics, including the occurrence and timing of flowering. These results provide context for the association observed in the present study, although the variable used here represents flowering levels derived from images rather than a direct count of flowers.

**Figure 6:**
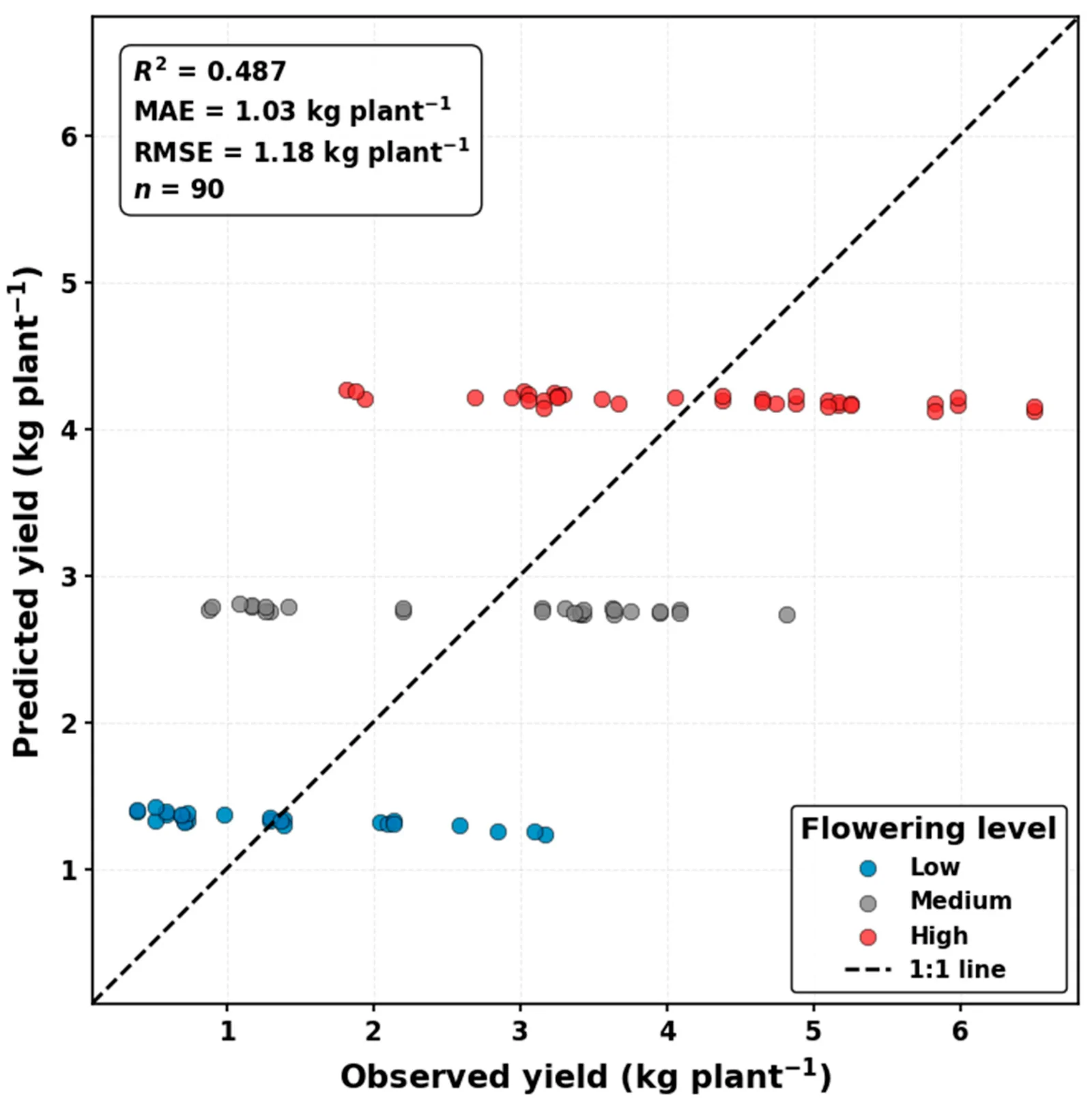
Cross-validation performance for the combined dataset (Arara + Mundo Novo), showing R^2^, MAE, and RMSE.

Cross-validation provided an additional assessment of the consistency of this relationship across different data partitions. For the combined dataset, the mean R^2^ across resamplings was 0.487, whereas the pooled out-of-sample predictions resulted in R^2^ = 0.489, MAE = 1.03 kg plant^−1^, and RMSE = 1.18 kg plant^−1^ (Figure 6). The pooled R^2^ was broadly consistent with the mean cross-validation R^2^, indicating that the predictive relationship was observed across the different data partitions. Nevertheless, the variability among resamplings indicates that the strength of the relationship varied across subsets of the data. These results also suggest that flowering level alone does not account for all variation in plant yield.

This interpretation is also consistent with the complexity of the relationship between flowering and fruit development in coffee plants. Ref. [21] in a review of *Coffea arabica* phenology, highlighted that yield formation involves several stages between flowering and fruit development and is influenced by plant characteristics and environmental conditions throughout the production cycle. Thus, although greater flowering intensity may be associated with higher yield, flowering intensity does not independently determine final yield. This context helps explain why, in the present study, flowering level showed a relevant association with yield but did not account for all of the observed variability.

**Figure 7:**
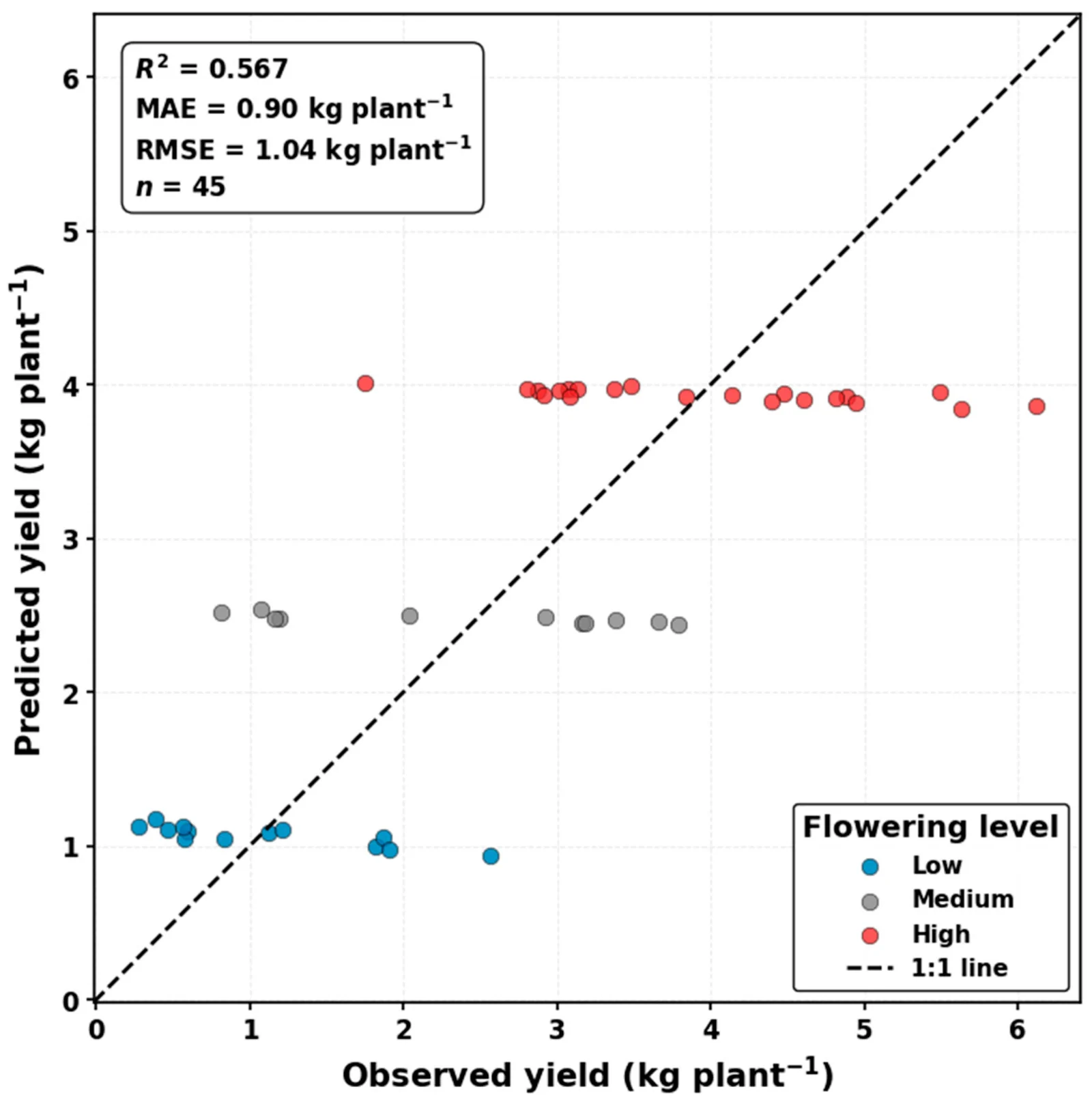
Cross-validation performance for the Arara cultivar, showing R^2^, MAE, and RMSE.

When the cultivars were analyzed separately, distinct patterns were observed. For Arara, the mean R^2^ from cross-validation was 0.497, whereas the pooled out-of-sample predictions presented R^2^ = 0.567, MAE = 0.90 kg plant^−1^, and RMSE = 1.04 kg plant^−1^ (Figure 7). These results indicate that flowering level was associated with a substantial proportion of the observed variation in yield for this cultivar, although variability among cross-validation resamplings was also observed.

**Figure 8:**
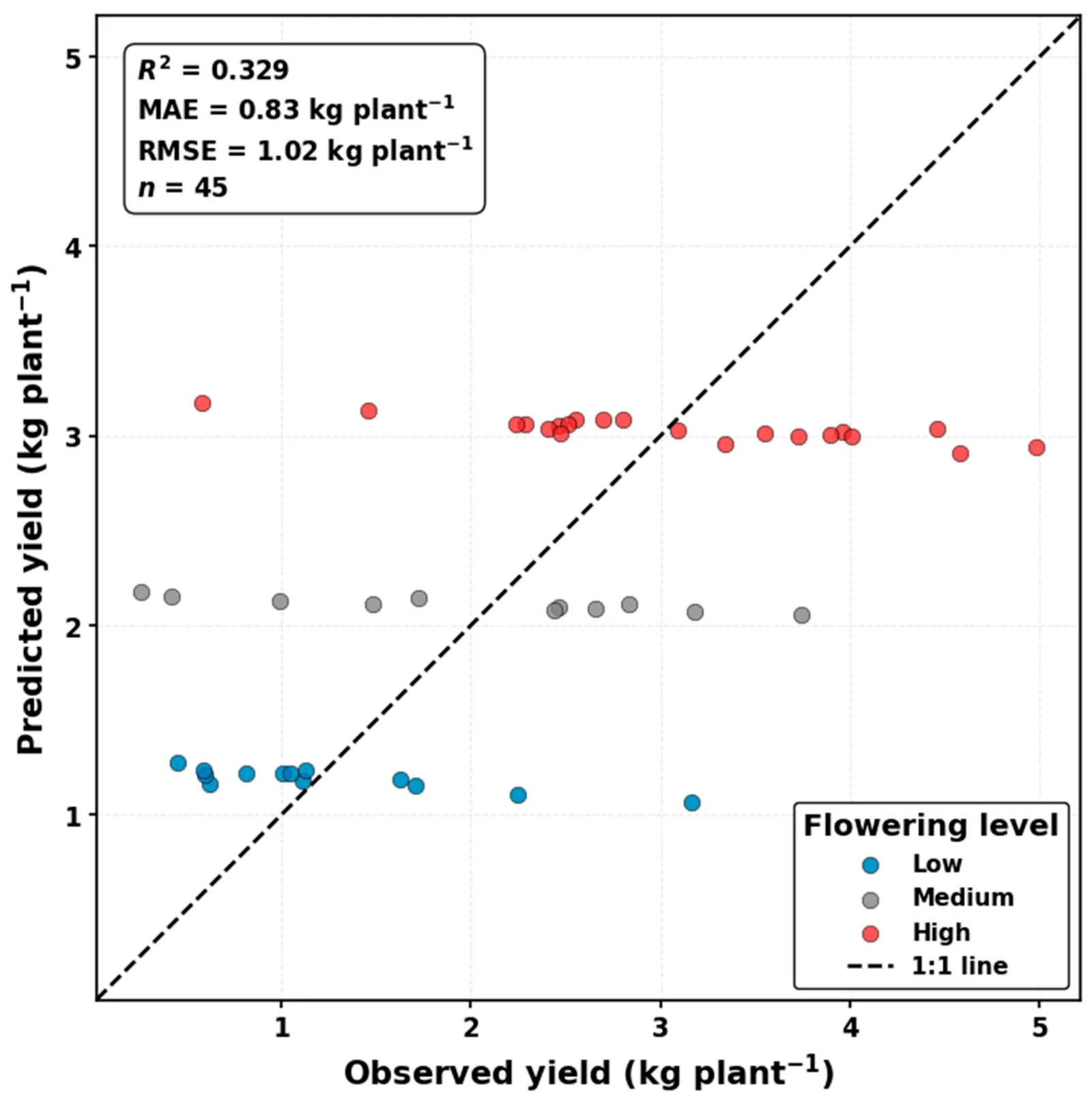
Cross-validation performance for the Mundo Novo cultivar, showing R^2^, MAE, and RMSE.

For Mundo Novo, the mean R^2^ from cross-validation was 0.210, whereas the pooled out-of-sample predictions presented R^2^ = 0.329, MAE = 0.83 kg plant^−1^, and RMSE = 1.02 kg plant^−1^ (Figure 8). The lower R^2^ indicates that flowering level accounted for a smaller proportion of the observed yield variability in Mundo Novo compared with Arara. However, the prediction errors were similar between the two cultivars, with MAE and RMSE of 0.83 and 1.02 kg plant^−1^, respectively, for Mundo Novo. These results, together with the significant flowering level × cultivar interaction, indicate that the magnitude of the association between the image-derived flowering variable and yield differed between the two cultivars.

The difference observed between Arara and Mundo Novo is relevant for interpreting the ability of the flowering variable to represent yield. In Arara, the higher R^2^ values from the pooled predictions indicated a stronger association between the variables, whereas Mundo Novo showed a lower proportion of yield variability represented by the flowering variable. Ref. [4] demonstrated that coffee yield may exhibit different relationships with phenological characteristics under different growing conditions and harvests, reinforcing that the relationship between flowering and yield should be interpreted considering the variability present in the production system. Similarly, ref. [22] reported differences in the flowering-to-maturation period among Arabica coffee cultivars, indicating that phenological responses may vary according to the genetic material and production cycle.

In this context, differences in flowering variability and synchronization between cultivars may have contributed to the weaker association between the flowering proxy and subsequent yield in Mundo Novo. Flowering events distributed over different periods may result in fruits at different developmental stages, increasing maturation heterogeneity and potentially reducing the direct association between flowering intensity and final yield, a behavior also described by [23,24].

In this study, flowering level was obtained from a variable derived from brightness characteristics in RGB images and used to classify plants into three levels: low, medium, and high. Therefore, the statistical relationship observed should be interpreted as an association between this image-derived variable and yield, rather than as a direct measure of the number of flowers present on the plants. Nevertheless, the results indicated that this information was associated with a relevant portion of the observed yield variation, particularly for the combined dataset and the Arara cultivar.

The use of RGB images to represent flowering-related characteristics is supported by previous studies. Ref. [14] developed an approach to quantify coffee flowering using RGB images acquired by UAV and found a Spearman correlation of ρ = 0.761 between the floral area identified in the images and the number of flowers determined in the field (*p* < 0.001). The authors therefore demonstrated that flowering-related information can be extracted from RGB images acquired using aerial platforms. Although the approach used in the present study differs, being based on the classification of plants into flowering levels rather than the direct estimation of flower number, the results are consistent regarding the possibility of obtaining information related to coffee flowering from RGB images.

**Figure 9:**
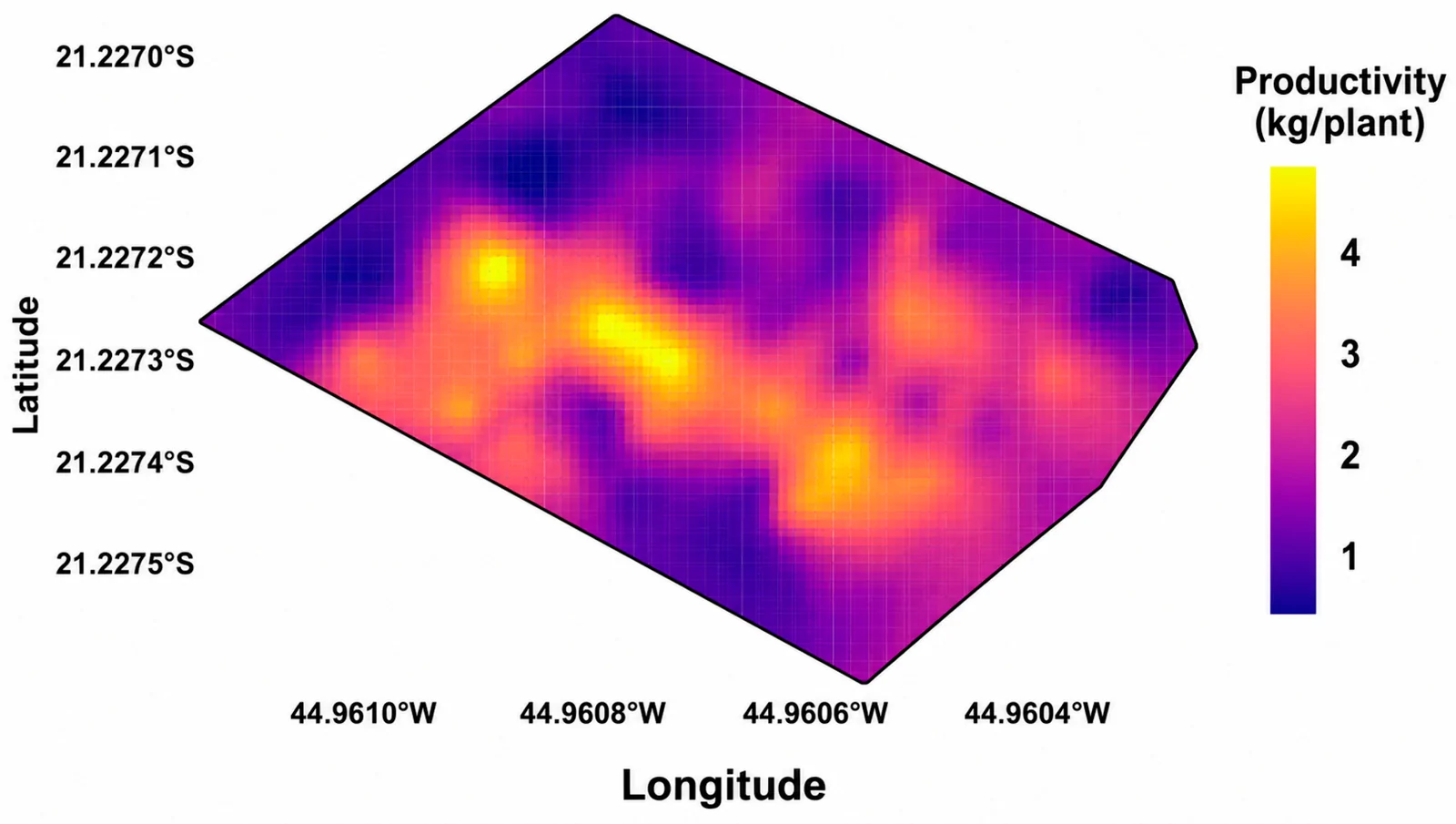
Spatial distribution of observed yield.

The spatial analysis (Figure 9) complemented the statistical results by showing how yield and flowering level were distributed within the experimental area. The observed yield map showed a structured spatial distribution, with higher values concentrated mainly in the central region of the area, whereas lower values occurred more frequently in the peripheral regions. This pattern indicates that yield exhibited spatial organization within the evaluated area rather than an apparently random distribution among plants.

The spatial distribution of flowering level showed a pattern similar to that observed for yield. Regions with a higher concentration of plants classified as having a high flowering level largely coincided with areas that presented higher yield values. Likewise, regions characterized by low flowering levels showed a greater occurrence of plants with lower yield. The medium level showed an intermediate distribution, occurring mainly in transition zones between areas with higher and lower yield.

**Figure 10:**
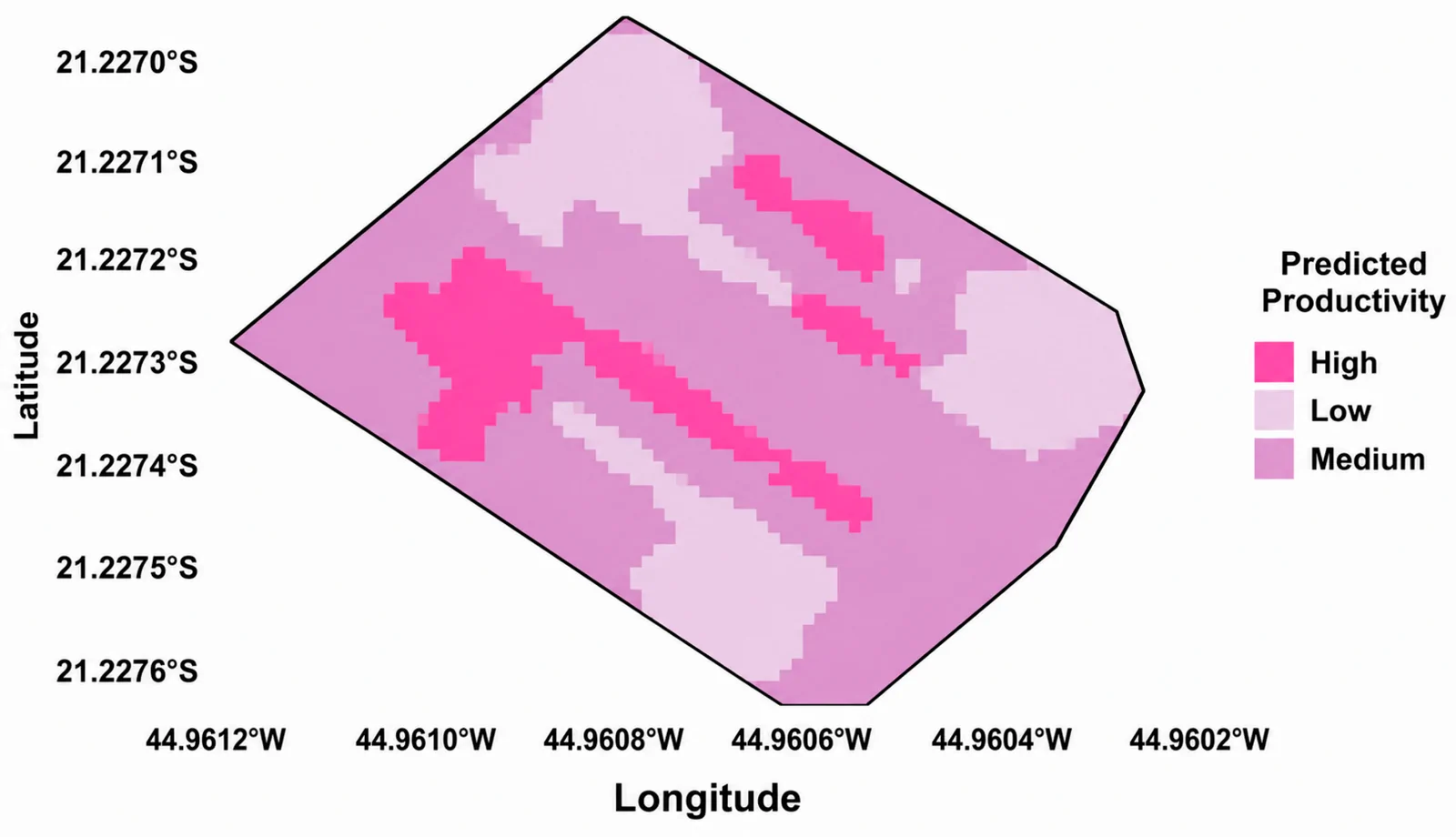
Spatial distribution of the productive potential estimated from flowering intensity classified by the Deep Learning model.

The comparison between observed yield and the spatial distribution of flowering levels reinforced this correspondence between the patterns (Figure 10). Areas with a higher concentration of plants classified as having a high flowering level also showed a greater concentration of plants with high yield, whereas areas predominantly classified as low were associated with lower yield values. This result indicates that the spatial information derived from RGB images was able to represent part of the spatial heterogeneity subsequently observed in yield across the experimental area.

Results obtained using other characteristics derived from aerial images also demonstrate the ability of remote sensing to represent variability within coffee plantations. Ref. [25] using RGB images acquired by UAV in an experimental coffee area in Minas Gerais, demonstrated the potential of these images for spatial monitoring of the crop, revealing variability in RGB indices throughout the production cycle. In a separate study, ref. [25] used RGB images acquired by UAV together with characteristics such as leaf area index, plant height, and canopy diameter to develop coffee yield estimation models in Minas Gerais. Leaf area index and canopy diameter were among the most important characteristics for yield prediction. These results support the use of information derived from aerial images as spatialized indicators of crop performance, although the variable used in the present study is different.

The concentration of higher yield values in the central region and lower values in the peripheral areas suggests a possible influence of spatial heterogeneity in conditions within the cultivated area. From an agronomic perspective, the continuity of higher-yield zones in the central region may be related to spatially distributed factors such as water availability, soil fertility, microclimatic conditions, and plant vigor. Likewise, the greater fragmentation observed in peripheral regions may be associated with edge effects and greater heterogeneity of environmental conditions, factors frequently related to greater yield variability in agricultural areas [21,26]. However, because these variables were not jointly evaluated in the present study, these interpretations should be considered only as possibilities, and causal relationships cannot be established. Ref. [4] demonstrated that Arabica coffee yield in different regions of Minas Gerais was related not only to flowering occurrence but also to climatic conditions during different stages of the production cycle, particularly during fruit development. Therefore, the spatial organization observed in the present study may reflect the combined influence of different factors, although it is not possible to determine which factors effectively contributed to the identified pattern.

The results show that the flowering variable derived from RGB images was positively associated with yield and that this relationship was also manifested spatially within the experimental area. Cross-validation demonstrated, however, that the magnitude of this relationship varied between cultivars, with greater consistency for Arara and greater dispersion for Mundo Novo. These results indicate that the relationship between the information obtained from the images and yield was not uniform across the evaluated cultivars. The combination of correlation, regression, cross-validation, and spatial analysis therefore allowed not only the statistical association between the variables to be evaluated, but also their spatial distribution and the consistency of this relationship across different data partitions and groups of plants.

From a Precision Agriculture perspective, the results indicate that RGB images acquired by UAVs can provide a spatialized variable associated with coffee yield. The automatic classification of flowering levels makes it possible to transform information obtained from images into classes that can be spatially related to plant yield performance. This possibility is consistent with the results of [25], who demonstrated the potential of RGB images acquired by UAVs for spatial monitoring of coffee plantations and yield estimation based on image-derived characteristics. In this context, the approach may assist in the early identification of areas with different patterns associated with yield performance and, consequently, support more targeted monitoring and management strategies. However, operational application of this approach requires further evaluation across different production cycles, cultivars, and growing conditions to assess the stability of the observed relationship and its applicability under different field conditions.

## 4. Conclusions

This study evaluated the use of RGB images acquired by remotely piloted aircraft (RPA) combined with Deep Learning models to classify coffee plants according to three levels of a flowering proxy. Among the evaluated architectures, ResNet50V2 was selected for final evaluation based on its validation performance, achieving 92.12% accuracy, 92.40% macro-precision, 91.81% macro-recall, and 92.06% macro-F1 on the test set. These results indicate that the proposed image-based approach can discriminate the defined flowering levels from RGB images.

The flowering proxy showed a positive association with field-observed yield. For the combined dataset of Arara and Mundo Novo, Spearman’s correlation was ρ = 0.719 (*p* < 0.001), while the ordinal regression model resulted in R^2^ = 0.535, with an estimated increase of 5.60 kg plant^−1^ per increment in flowering level. In the consolidated out-of-sample predictions, the model achieved R^2^ = 0.515, MAE = 3.82 kg plant^−1^, and RMSE = 4.39 kg plant^−1^. The spatial analysis also showed correspondence between the distribution of flowering levels derived from RGB images and the subsequent spatial distribution of yield, with higher flowering levels generally coinciding with areas of higher productivity.

The findings indicate that RPA-based RGB imaging and Deep Learning can provide spatialized information related to coffee flowering and its subsequent productive performance, with potential applications in precision agriculture. However, differences in predictive performance between cultivars and variability across cross-validation partitions indicate that the flowering proxy should be considered as one source of information rather than a standalone representation of yield. Further studies across production cycles, cultivars, and environmental conditions, together with complementary spectral, climatic, physiological, and agronomic variables, may improve the robustness and generalization of the approach.

## Figures and Tables

**Table 1:** Summary of the evaluated architectures and their classification layers.

Model	Input	Main Configuration	Training	LR
Thresholding/counting baseline	White-pixel count	Threshold 215; 3 equal-width classes	Deterministic	—
Custom CNN	RGB 400 × 200 × 3	4 convolutional blocks; GAP; Dense 256–128; 3-class softmax	Adam	0.001
VGG16	RGB 400 × 200 × 3	ImageNet pretrained; last 8 layers trainable; GAP; Dense 512–256; 3-class softmax	Transfer learning + Adam	0.0001
MobileNetV2	RGB 400 × 200 × 3	ImageNet pretrained; last 30 layers trainable; GAP; Dense 256; 3-class softmax	Transfer learning + Adam	0.0001
ResNet50V2	RGB 400 × 200 × 3	ImageNet pretrained; Dense 256; 3-class softmax; last 50 layers fine-tuned	Two-stage transfer learning	0.001/0.00001

**Table 2:** Agreement among proxy flowering-intensity classes obtained using different threshold values.

Threshold Comparison	Agreement (%)	Cohen’s Kappa
210 vs. 215	94.22	0.908
215 vs. 220	93.72	0.900
210 vs. 220	87.95	0.809

**Table 3:** Performance of the Deep Learning architectures in classifying the flowering proxy on the validation set.

Model	Validation Accuracy	Macro Precision	Macro Recall	Macro F1-Score	Loss
ResNet50V2	0.9454	0.9411	0.9479	0.9443	0.1342
MobileNetV2	0.9328	0.9280	0.9334	0.9305	0.2246
Custom CNN	0.8529	0.8397	0.8884	0.8467	0.3619
VGG16	0.8319	0.8229	0.8614	0.8332	0.4381

## Data Availability

The data presented in this study are available from the corresponding author upon reasonable request. The data are not publicly available at this time, as they are still being analyzed in conjunction with additional datasets from the same study area for the preparation of further publications.
